# Surgical Phase Duration in Robot-Assisted Partial Nephrectomy: A Surgical Data Science Exploration for Clinical Relevance

**DOI:** 10.3390/diagnostics13213386

**Published:** 2023-11-05

**Authors:** Pieter De Backer, Maria Peraire Lores, Meret Demuynck, Federico Piramide, Jente Simoens, Tim Oosterlinck, Wouter Bogaert, Chi Victor Shan, Karel Van Regemorter, Aube Wastyn, Enrico Checcucci, Charlotte Debbaut, Charles Van Praet, Rui Farinha, Ruben De Groote, Anthony Gallagher, Karel Decaestecker, Alexandre Mottrie

**Affiliations:** 1 ORSI Academy, 9090 Melle, Belgium; 2 IbiTech-Biommeda, Department of Electronics and Information Systems, Faculty of Engineering and Architecture, Ghent University, 9000 Ghent, Belgium; 3 Department of Human Structure and Repair, Faculty of Medicine and Health Sciences, Ghent University, 9000 Ghent, Belgiumcharles.vanpraet@uzgent.be (C.V.P.);; 4 Young Academic Urologist—Urotechnology Working Group, NL-6803 Arnhem, The Netherlands; 5 Department of Urology, ERN eUROGEN Accredited Centre, Ghent University Hospital, 9000 Ghent, Belgium; 6 Department of Surgery, Candiolo Cancer Institute, FPO-IRCCS, 10060 Turin, Italy; 7 Faculty of Medicine, KU Leuven, 3000 Leuven, Belgium; 8 Department of Urology, Onze-Lieve Vrouwziekenhuis Hospital, 9300 Aalst, Belgium; 9 Department of Urology, AZ Maria Middelares Hospital, 9000 Ghent, Belgium

**Keywords:** phase duration assessment, partial nephrectomy, video analysis, surgical data science

## Abstract

(1) Background: Surgical phases form the basic building blocks for surgical skill assessment, feedback, and teaching. The phase duration itself and its correlation with clinical parameters at diagnosis have not yet been investigated. Novel commercial platforms provide phase indications but have not been assessed for accuracy yet. (2) Methods: We assessed 100 robot-assisted partial nephrectomy videos for phase durations based on previously defined proficiency metrics. We developed an annotation framework and subsequently compared our annotations to an existing commercial solution (Touch Surgery, Medtronic™). We subsequently explored clinical correlations between phase durations and parameters derived from diagnosis and treatment. (3) Results: An objective and uniform phase assessment requires precise definitions derived from an iterative revision process. A comparison to a commercial solution shows large differences in definitions across phases. BMI and the duration of renal tumor identification are positively correlated, as are tumor complexity and both tumor excision and renorrhaphy duration. (4) Conclusions: The surgical phase duration can be correlated with certain clinical outcomes. Further research should investigate whether the retrieved correlations are also clinically meaningful. This requires an increase in dataset sizes and facilitation through intelligent computer vision algorithms. Commercial platforms can facilitate this dataset expansion and help unlock the full potential, provided that the phase annotation details are disclosed.

## 1. Introduction

Surgical video recordings have become an indispensable tool in surgery, and their uses can be categorized into three main groups [1]:

1. Surgical Education and Training: Video recordings provide a valuable resource for trainees to observe and learn surgical procedures from experts [2,3]. By capturing critical moments during surgery, videos also allow for retrospective analysis and discussions among surgical teams and aid in complex decision making. Literature reports have documented the utility of surgical video review in assessing anatomical landmarks [4], identifying surgical errors, refining surgical techniques [5], and the development [6] and assessment [7] of objective surgical skill metrics, which can be used for training. Additionally, video-based platforms [8] and telemedicine applications have enabled remote surgical education and mentorship [9], further expanding access [10] to surgical training opportunities.
2. Patient Outcome Assessment: Surgical video recordings provide objective documentation of surgical procedures, enabling a detailed analysis of surgical techniques, complications, and their impact on patient outcomes. Retrospective video analysis has been utilized in several studies to investigate patient outcomes [11].
3. Quality Improvement Initiatives: By reviewing surgical videos, surgical teams can identify areas for improvement, refine surgical approaches, evaluate adherence to established protocols, and enhance patient safety [12]. Furthermore, video-based quality assurance programs have been implemented to monitor surgical performance, benchmark outcomes, and ensure standardized practices across institutions [13].

Uniform surgical phase definitions and assessments are enabling factors in many of the applications above. Surgical phase assessment allows for consistent and standardized indexing across procedures, which facilitates video review, analysis, and sharing. As video analysis, computer vision, artificial intelligence, and data analytics (so-called ‘surgical data science’) enter surgical theaters, the importance of correct and uniformly defined surgical phases increases [14]. This is especially true for complex surgeries that require decomposition into simpler phase blocks for objective assessment and review [15]. Previous work has also shown that phase definitions, even for non-complex procedures, are often not uniformly defined [16], and there is a clear need for common ontologies to unlock the full potential of surgical data science [17].

Up to now, researchers have been focusing on shorter and often linear procedures such as laparoscopic cholecystectomy [18], whilst more varying and complex procedures, which might benefit most from new data science insights, remain poorly investigated.

One such longer procedure with more inherent variation is robot-assisted partial nephrectomy (RAPN). Partial nephrectomy itself is the gold-standard treatment for patients with T1 renal cell carcinoma [19]. RAPN has become increasingly popular, amongst others, because of its low morbidity and early convalescence when compared to open surgery [20] and its shorter learning curve when compared to laparoscopy [21].

Apart from the usefulness of surgical phase indexing for video manipulation and case revision, surgical errors are often related to, and dependent on, specific surgical phases [22]. As such, surgical phases form an initial and crucial step toward achieving automated and objective surgical skill assessment. A skill assessment system should first identify the phase it is in before focusing on the errors inherent to that phase. Error reduction and skill enhancement through proficiency-based progression, as compared to classical training, have proven to be beneficial for surgical skill acquisition and quantification [23]. Surgical phase duration has not yet been shown to correlate with surgical skill. When assessing clinical outcomes, the full surgical duration has been investigated for correlations [24,25,26]. Apart from outcomes, retrieving correlations between diagnostic parameters and phase durations also has logistic benefits, as they could, for instance, enable a more precise OR time planning estimation at diagnosis. To our best knowledge, the analysis of the specific surgical phase duration and possible correlations with patient-specific clinical parameters at diagnosis or after treatment has not been performed.

In this work, we first proposed a vision-based framework for objective, uniform, and precise surgical phase definitions during RAPN. The definitions are based on previously developed and clinically validated metrics for a proficiency-based curriculum [7].

Secondly, we manually analyzed 100 RAPN procedures to refine and optimize the previously defined visual cues. We compared our annotations to a commercially available online platform (Touch Surgery™—Medtronic, Minneapolis, MN, USA – version 22 February 2023), which provides phase information when uploading videos to its online library without user input. We evaluated its correctness by comparing manually performed in-house annotations.

Finally, we performed the first statistical exploration of phase durations and patient characteristics/clinical outcomes in RAPN.

## 2. Materials and Methods

### 2.1. Data Collection and Visual Cue Definition

From January 2018 to June 2022, 100 transperitoneal RAPN procedures were retrospectively and randomly collected from an existing video database in OLV Aalst Hospital (80 procedures) and Ghent University Hospital (20 procedures) after IRB approval. All procedures were performed on Intuitive Xi robotic systems (Intuitive™, Sunnyvale, CA, USA). All procedures were either partially or completely performed by expert urologists, implying that procedural parts could have been performed by urologists in training, but always under expert supervision. Recordings were discarded if they did not contain a full-length procedure. Table 1 displays the patient characteristics of the 100 procedures included in the dataset.

We derived 16 visually distinctive phases from the validated RAPN metric framework developed by Farinha et al. [6,7]. We defined exact visual cues as starting points for surgical phases. Table A1 (Appendix A) depicts the exhaustive phase list and corresponding visual cues. We adapted the existing framework defined for left-sided RAPN for common phases present in both left- and right-sided RAPN. We refer to this annotation framework as the Orsi-framework.

**Figure A1:**
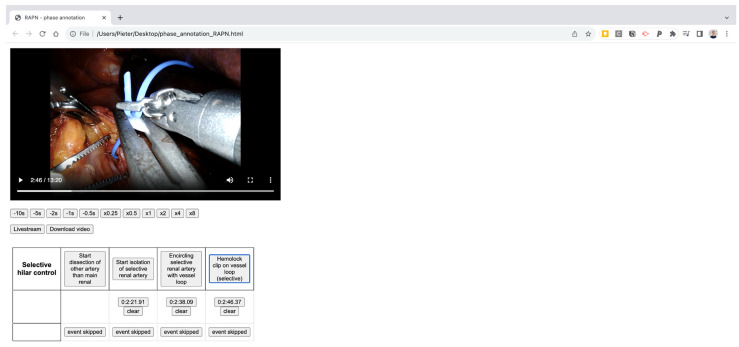
Screenshot of the in-house-developed HTML file for phase analysis.

An in-house-developed manual annotation tool was used, consisting of an in-browser viewer that allows one to load a video, precisely define the starting points of selected phases, and export the timepoints with millisecond precision afterward. An example of the manual annotation tool can be found in Appendix A (Figure A1). All videos were assessed by three medical students (MD, CVS, TO) after consensus on the visual definitions with a consultant urologist (RDG). The final manual student annotations were subsequently double-checked by a second consultant urologist (MPL).

### 2.2. Comparison to Commercial Software

The precise and more nuanced Orsi definitions were concatenated into 13 phases to be consistent with the Touch Surgery (TS) phase definitions for a one-to-one comparison. The phases’ consistency can be found in Table 2. Two medical students (KVR, AW) reviewed all videos for correctness after automated, rule-based concatenation.

All uploaded videos were automatically anonymized with the removal of all possible identity clues, which included the removal of out-of-body segments, as well as the removal of segments with the TilePro™ function (Intuitive™, Sunnyvale, CA, USA), while they display patient-specific information such as CT, MRI, or 3D model data [27]. Anonymization did not alter the case durations, as these parts were blacked out rather than cropped. Subsequently, videos were uploaded to the TS platform (software version February 2023). The Orsi analysis was performed on fully non-anonymized videos under IRB approval.

After upload and processing, TS timepoints were manually extracted from the online commercial platform.

The Touch Surgery platform does not provide details on the phase definition rules. The videos underwent an automated similarity analysis in which the manual Orsi annotations were compared to Touch Surgery annotations by using a predefined loss function. This function objectifies how different phases were annotated and detects procedures that have the least similarity.

**Figure 1:**
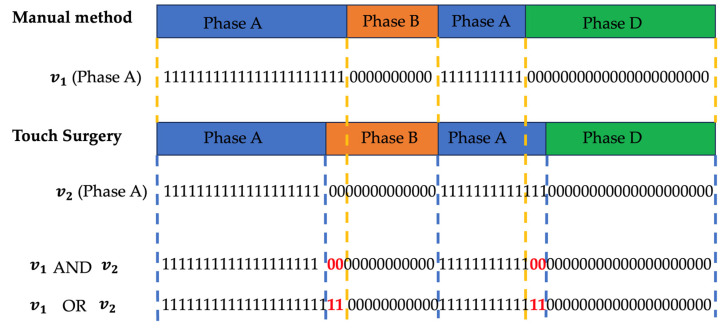
Example of the IoU calculation method. Depicted in red are the differences picked up by the nominator and denominator in the IoU formula to better quantify the overlap or difference in recurring phases.

We base the loss function on a commonly used metric called Intersection-over-Union (IoU). For each phase, a binary vector is created, which indicates 1 for seconds that belonged to that phase for the given annotation method and 0 elsewhere. The total sum of all phase vectors equals the total procedure time in seconds. The concept is visualized in Figure 1. The IOU per phase is then calculated as follows:

$$IoU\left(phase\;X\right)=\frac{sum\left(v_{1}\;AND\;v_{2}\right)}{sum\left(v_{1}\;OR\;v_{2}\right)}$$

where $v_{1}$ is the vector with the number of seconds spent in a certain phase X in the manual annotation, and $v_{2}$ is the vector with the number of seconds spent in phase X according to the Touch Surgery annotation. Both are linked in a one-to-one temporal fashion, as depicted in Figure 1.

As such, the numerator holds the number of seconds where both procedures ‘agree’ on the investigated phase for the full procedure. The overlaps are summed, as phases can reoccur. The denominator counts the number of seconds where the phase was indicated by at least one of the two annotation methods. A perfect correspondence between both annotation approaches results in an $IoU\left(phase\right)$ of 100%. To determine the final loss, we multiply all $IoU\left(phase\right)$ and take the negative logarithm.

The loss function is described below:

$$Loss=-\ln(\underset{phases}{\prod }IoU(phase))$$

### 2.3. Clinical Analysis

We explored correlations between phase durations and the following clinical parameters: age, gender, tumor complexity (PADUA score), tumor size, prior abdominal surgery, BMI, pT stage, histology, preoperative and postoperative renal function as measured by eGFR, and creatinine. We also examined postoperative complications. Durations were assessed for both TS and manual (M) definitions. The correlation examination was performed by using Spearman’s and Kendall’s tau tests, as appropriate. In the case of a statistically significant correlation, a linear regression model was then built to analyze the relationship between the identified variables. All of the analyses were performed by using Jamovi software v2.3.

## 3. Results

### 3.1. General Phase Data Analysis and Comparison

Touch Surgery does not provide a phase assessment in the case of cystic renal lesions, despite it being a similar technical approach. As such, the resulting final dataset for the side-by-side comparison was reduced to a total of 86 RAPN procedures. Table 3 shows the descriptive statistics for our concatenated manual (M) phase analysis and the TS analysis for the 86 analyzed procedures.

**Figure 2:**
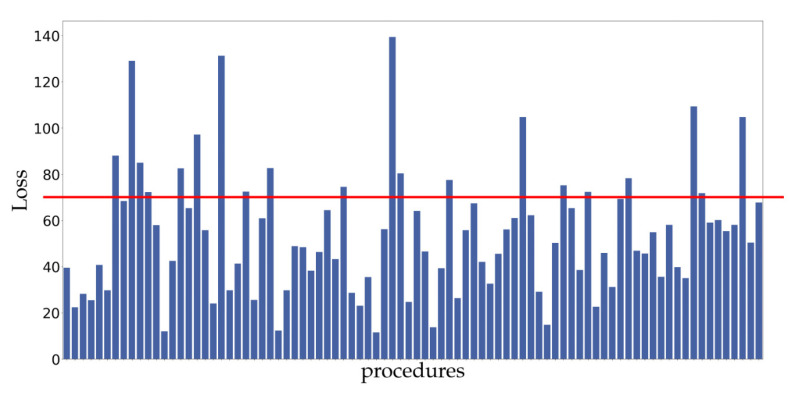
Loss for all 86 analyzed procedures. Twenty procedures scored above the empirically set threshold of 70 and were analyzed for discrepancies in definition and labeling.

At first sight, the phase durations on both platforms are quite different. When looking at the similarity between phase definitions in the full dataset, the 20 RAPN procedures with the least similarity (consistent with a loss higher than 70) were analyzed in depth for phase assessment differences. Figure 2 shows the loss for all procedures.

The following recurring discrepancies were withheld in poorly corresponding videos. The ‘Identification of Anatomical Landmarks’ phase mainly shows overlap with the ‘Colon Mobilization’ phase; i.e., ‘Identification of Anatomical Landmarks’ is defined by one annotation method, whereas ‘Colon Mobilization’ is defined by the other annotation method at this timepoint. This occurs in both ways and is perhaps because this phase is poorly defined and allows for multiple interpretations. Furthermore, although port insertion and surgical access were not always recorded, it was not annotated in TS in half of the cases, leading to a longer duration of ‘Colon Mobilization’. From ‘Colon Mobilization’ onwards, we do note that, in general, the same number of phases are annotated. A clearly differing visual definition is present in ‘Kidney mobilization’ and ‘Tumor identification’. Ultrasound use and guided demarcation form an important part of tumor identification, and in certain procedures, ultrasound (US) is initiated before the opening of Gerota’s fascia. In the manual phase analysis, this US usage is considered a subphase of ‘Tumor identification’ and thus marks the beginning of this phase. Touch Surgery does not always recognize US usage as part of ‘Tumor identification’ yet, resulting in a missed phase initiation. Touch Surgery recognizes the start of the ‘Tumor identification’ phase when US is used for the second time when the renal parenchyma with the tumor zone is already freed from perirenal fat. This also results in longer ‘Tumor Identification’ phases in the manual assessment. As the manual assessment is more nuanced and the definition is different, a side-by-side comparison of accuracy is irrelevant. This differing definition impacts all phase durations before the onset of hilar clamping and tumor excision, which makes the phase duration comparison for other phases before ‘Hilar Clamping’ unreliable.

We do note that when combining all surgical manipulations before ‘Hilar Clamping’, which is the combination of ‘Port Insertion and Surgical Access’, ‘Colon Mobilization’, ‘Identification of Anatomical Landmarks’, ‘Hilar Dissection’, and ‘Kidney Mobilization’, very similar mean and median durations are observed (means are 76.5 and 76.3 min, medians are 62 and 64.9 min for respectively M and TS assessment).

The next important surgical landmark entails tumor excision, which is most often performed after hilar clamping. We see here that of all 100 procedures, 77 procedures were performed off-clamp. The decision for off-clamp tumor excision is made by the expert surgeon on a case-by-case basis and was not a criterion for video case selection. Touch Surgery did not indicate tumor excision and tumor retrieval in two cases due to our anonymization protocol. Both cases had patient-specific CT scan or 3D model info in the console view, which was anonymized before uploading. Despite being a clear drawback for comparison, these phases should not impact median times, where we note similar trends. The combined duration of ‘Hilar clamping’ and ‘Tumor Excision’ is very similar between the two methods. We find mean and median combined values of, respectively, 9.2 and 6.9 min for the manual assessment and 9.9 and 8.6 min for the TS assessment. The main factors contributing to the longer ‘Tumor Excision’ times in TS are twofold. TS includes the application of hemostatic agents, which is excluded in our manual assessment, and the TS ‘Tumor Excision’ phase starts as soon as the kidney is manipulated after clamping, whereas, in our assessment, it only starts when blunt dissection takes place. This also results in a longer manual ‘Hilar Clamping’ duration.

After tumor excision, we enter the last procedural part, in which the kidney is reconstructed after specimen retrieval. ‘Specimen Retrieval’ has very similar durations; nonetheless, we note that, despite our best efforts, both our manual assessment and TS missed annotating one specimen retrieval, albeit in different procedures. Similar to ‘Hilar Clamping’, the ‘Hilar Unclamping’ phase is once again shorter in TS. The main contributing factor is the renorrhaphy starting point definition. In TS, this phase starts when needles are brought close to the parenchyma, and thus, this ends the unclamping. In our assessment, this phase only starts at the first parenchymal suture. This is also reflected in shorter renorrhaphy times for our assessment and confirms the narrower definition. ‘Specimen retrieval and Closure’ has a similar time range and includes renal retroperitonealization. The ‘Operation Finished’ definition is more precise in our assessment and marks when the camera is removed from the abdomen for the last time, whilst for Touch Surgery, this is simply the end of the video, resulting in an empty phase duration.

### 3.2. Clinical Data Correlation Exploration

Prior abdominal surgery did not significantly impact any of the phases’ durations, independent of the phase assessment methodology (TS or M). Likewise, no significant impact was found for age, gender, pT stage, histology type, or pre-/postoperative renal function. The postoperative complication rate was too low to find any correlations.

**Figure 3:**
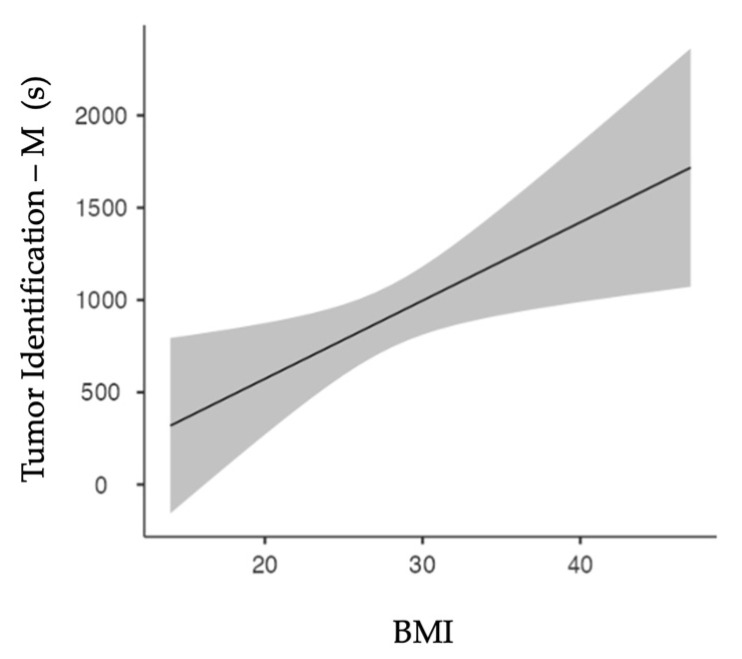
Linear regression model for BMI and surgical phase duration of manual tumor identification. Increasing BMI correlates with increasing tumor identification duration.

Spearman’s test showed a significant correlation (*p* = 0.011) between patients’ BMI and the ‘Tumor Identification’ duration for manually assessed cases only. Figure 3 shows the linear regression model. Increased BMI results in increased ‘Tumor Identification’ periods (*p* = 0.011).

**Figure 4:**
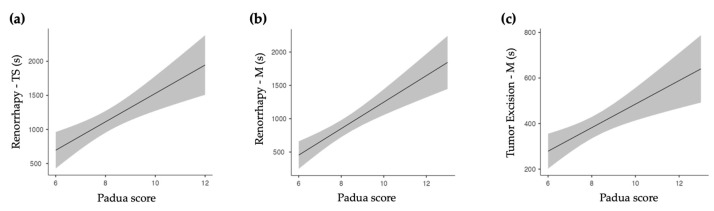
Linear regression model for PADUA scores. (**a**) Positive correlation with ‘Renorrhaphy’ as assessed by Touch Surgery (TS). (**b**) Positive correlation with ‘Renorrhaphy’ as assessed manually (M). (**c**) Positive correlation with ‘Tumor Excision’ duration when assessed manually (M).

When assessing tumor complexity according to the PADUA scores, we see that the ‘Renorrhaphy’ duration has a significant positive correlation with the PADUA score (*p* < 0.001 for both Touch Surgery and manual assessment). Likewise, ‘Tumor Excision’ was significantly correlated with the PADUA score, but only in the manual assessment (*p* < 0.001). In the linear regression, the PADUA score confirmed a positive correlation with the ‘Renorrhaphy’ duration (*p* < 0.001 for both TS and M assessments, Figure 4a and Figure 4b, respectively). Figure 4c depicts the positive correlation between the manual assessment of ‘Tumor Excision’ and the PADUA score (*p* < 0.001).

## 4. Discussion

### 4.1. Data Quality and Visual Clue Definition

The retrospective data collection poses a limitation on data quality. As both hospitals have residency and fellowship programs, procedural parts have been performed by non-experts, which might bias the results. Previous work has also shown that the console time significantly decreases whilst going through the learning curve for off-clamp RAPN [28]. Nonetheless, these non-expert procedural parts were not documented at the time of surgery.

Defining visual clues for phase analysis is a repetitive process in which the multicentric approach allowed for a more robust and precise definition. Several iterations were required before agreeing on a final template, as provided in Table A1, which was then used for the full dataset investigation. Despite our best efforts in checking all procedures fivefold, post hoc data analysis revealed that we still missed annotating one phase. As such, phase annotation is a time-intensive effort, which reveals the clinical need for intelligent systems that can support this automatically and consistently.

### 4.2. Platform Comparison

When comparing our manual platform and the Touch Surgery platform for RAPN phase assessment, we identify three key items.

Firstly, the lack of analysis of renal cysts in Touch Surgery is an apparent drawback, as it immediately discards 14% of the cases, despite having an identical surgical technique and approach.

Secondly, when analyzing both platforms for the remaining 86 procedures, we identified the lack of uniform and transparent definitions as the main inhibitor of an in-depth side-by-side comparison. Touch Surgery does not provide details on the phase analysis process. It is unknown whether the assessment happens fully autonomously, semi-autonomously, or fully manually. In the third case, nothing is known about the expertise level of the annotators, their training, or their interrater reliability. The platform provides no information on the used visual phase cues. On the other hand, no public consensus exists on objectively defined visual phase cues. Despite having based our definition on a published consensus for phase metrics, defining visual cues requires more details, and as such, there is a clear mismatch between the two definitions.

Thirdly, the assessment of the most dissimilar procedures, as identified by the loss function, shows that phase combinations and the evaluation of specific subphases result in greater similarity. As such, large surgical entities are annotated in a similar fashion. When assessing the full procedure, three major operative parts (preparation for tumor excision, tumor excision, and hemostasis and closure) were found to be similar. Nevertheless, they are insufficiently granular for immediate outcome correlation research.

### 4.3. Clinical Outcomes

BMI and the ‘Tumor Identification’ duration are positively correlated, which could be explained by higher BMI often involving more intraabdominal fatty tissue. As the kidney is surrounded by perinephric fat, tumor identification on the renal surface can indeed be more tedious. This correlation was not withheld in the TS definitions. This can be attributed to the apparent TS definitions of only annotating ‘Tumor Identification’ when the kidney is already fully exposed. Another contributing factor is our removal of echography TilePro™ segments in the TS group for anonymization purposes. Nevertheless, these segments were often correctly picked up by the TS platform, starting when ultrasound entered the body and ending upon ultrasound removal.

When tumor complexity rises, e.g., because of its larger size or endophytic nature, as expected, the tumor excision duration also increases. Likewise, the ‘Renorrhaphy’ duration increases with tumor complexity. Big or deep tumors can indeed leave large or difficult-to-reach renal resection beds, which can make it more technically challenging to effectively reconstruct the kidney or obtain adequate hemostasis. Nonetheless, it is noteworthy that these correlations were only withheld in the manual assessment. The missing correlation for TS might be attributed to the broader ‘Tumor Excision’ definition, which includes hemostatic agent applications. Touch Surgery retrieved statistical significance with the renorrhaphy duration only. We do note that 2 out of 86 cases were irrelevant for tumor excision comparisons, given the pre-upload anonymization. Furthermore, the manual data analysis was performed on 14 more cases.

## 5. Conclusions

Assessing surgical phase durations for clinical relevance requires nuanced and granular phase descriptions and definitions, which can be derived from metric-based training curricula. We note that the nature of this work is hypothesis generation, without implying causation. Nevertheless, we found initial objective insights into how factors like BMI and tumor complexity, assessed at diagnosis, correlate with the surgical phase duration.

Metric-based definitions effectively resulted in more clinical correlations when compared to a commercial platform, where the main drawback in the latter was missing phase definition information. As such, this manuscript might act as a guide toward better standardization for future phase analysis projects in RAPN.

Lastly, this work can serve as the basis for automated phase detection in RAPN, where deep-learning computer vision algorithms automatically define surgical phases [16]. This, in turn, enables larger patient cohort investigations, without the need for time-intensive manual annotations [29]. Furthermore, automated phase detection acts as an enabler toward fully automated surgical scene understanding [30], which, in turn, unlocks a myriad of other possible clinical applications [31].

## Figures and Tables

**Table 1:** Patient characteristics. Means and standard deviations are displayed for age, PADUA score, pathology size, and renal function. Other numbers are absolute counts as well as percentages, as 100 patients were included. Side refers to the kidney that was affected and operated on. Abbreviations: RCC = Renal Cell Carcinoma; TCC = Transitional Cell Cancer; eGFR = estimated Glomerular Filtration Rate.

Age (Years)	64.63 (±11.13)
Sex	
male	71
female	29
PADUA score	8.05 (±1.79)
RENAL score	8.47 (±2.77)
Side	
right	53
left	47
Pathology size (mm)	34.32 (±24.96)
Final Pathology Stage	
T0	25
T1a	49
T1b	18
T2a	5
T2b	2
T3a	1
Histology	
clear-cell RCC	52
papillary RCC	17
oncocytoma	17
angiomyolipoma	6
chromophobic RCC	4
cyst	2
sarcoma	1
TCC	1
Preoperative renal function	
eGFR (mL/min)	75.48 (±16.26)
creatinine (mg/dL)	1.03 (±0.39)
Postoperative renal function	
eGFR (mL/min)	71.70 (±19.19)
creatinine (mg/dL)	1.07 (±0.55)

**Table 2:** Congruence with phases derived from ERUS and definitions by Touch Surgery.

Orsi Definitions	Touch Surgery Definitions
(1) Port Insertion and Surgical Access	(1) Port Insertion and Surgical Access
(2) Colon (and Spleen) Mobilization	(2) Colon Mobilization (3) Identification of Anatomical Landmarks
(3) Hilar Control General (4) Selective Hilar Control	(4) Hilar Dissection
(5) Kidney Mobilization	(5) Kidney Mobilization
(6) Tumor Identification	(6) Tumor Identification
(7) Hilar Clamping	(7) Hilar Clamping
(8) Tumor Excision	(8) Tumor Excision
(9) Specimen Retrieval	(9) Specimen Retrieval
(10) Inner Renorrhaphy	(10) Renorrhaphy
(11) Hilar Unclamping	(11) Hilar Unclamping
(12) Outer Renorrhaphy	(10) Renorrhaphy
(13) Specimen Removal and Closing	(12) Specimen Removal and Closure
(14) Instrument Removal	(12) Specimen Removal and Closure
(15) Retroperitonealization of the Kidney	(12) Specimen Removal and Closure
(16) End of Operation	(13) Operation Finished

**Table 3:** RAPN phase duration results for one-to-one comparison between manual annotations ‘M(86)’ and Touch Surgery ‘TS(86)’ for the 86 procedures analyzed by Touch Surgery, as well as the values for all 100 procedures manually assessed ‘M(100)’, which includes the ‘M(86)’. The mean, median, standard deviation (SD), and interquartile distance (IQD) duration in minutes are depicted in normal black font. The number of occurrences of an annotated phase, this is whether it was annotated by the method at hand (TS versus M), is depicted in italics.

	Mean (±SD) (Minutes) Number of Instances			Median (IQD) (Minutes) Number of Instances		
**RAPN Phase**	**M (86)**	**TS (86)**	**M (100)**	**M (86)**	**TS (86)**	**M (100)**
Port Insertion and Surgical Access	5.8 (±5.6) 65	8.9 (±7.4) 34	6.2 (±5.6) 77	3.7 (8.3) 65	6.7 (7.6) 34	5.2 (9.7) 77
Colon Mobilization	5.6 (±7.6) 61	11.6 (±7.9) 71	5.7 (±7.7) 70	2.7 (7.4) 61	10.0 (11.3) 71	2.7 (7.6) 70
Identification of Anatomical Landmarks	13.1 (±8.7) 49	4.6 (±3.7) 50	12.0 (±8.5) 58	11.9 (14.5) 49	4.0 (5.5) 50	9.8 (12.3) 58
Hilar Dissection	16.0 (±11.5) 76	14.3 (±10.6) 74	16.3 (±12.3) 88	14.0 (19.6) 76	11.0 (16.0) 74	14.4 (19.3) 88
Kidney Mobilization	20.4 (±15.0) 84	27.5 (±17.7) 84	20.4 (±14.6) 98	18.4 (20.5) 84	24.6 (27.1) 84	17.8 (19.2) 98
Tumor Identification	15.6 (±15.5) 85	9.4 (±6.8) 80	15.2 (±14.6) 99	11.3 (11.3) 85	8.6 (6.8) 80	11.6 (12.4) 99
Hilar Clamping	2.9 (±3.6) 67	1.2 (±1) 65	3.2 (±4.1) 77	1.6 (2.5) 67	0.8 (1.2) 65	1.8 (2.6) 77
Tumor Excision	6.3 (±3.6) 86	8.7 (±4.9) 84	6.5 (±4.1) 100	5.3 (4.2) 86	7.8 (5.9) 84	5.3 (4.1) 100
Specimen Retrieval	1.5 (±2.5) 85	1.7 (±3.5) 83	1.7 (±2.8) 99	0.8 (1.0) 85	0.6 (1.0) 83	0.8 (1.0) 99
Renorrhaphy	14.9 (±9.9) 82	20.9 (±12.3) 77	15.8 (±11.6) 93	12.9 (11.7) 82	19.1 (15.2) 77	13.5 (12.7) 93
Hilar Unclamping	4.5 (±6.1) 65	2.7 (±6.4) 67	5.0 (±6.9) 75	2.2 (3.5) 65	1.1 (1.6) 67	2.9 (4.0) 75
Specimen Removal and Closure	8.2 (±6.0) 82	9.3 (±8.1) 71	8.5 (±6.3) 94	6.9 (6.1) 82	7.1 (8.5) 71	7.4 (6.5) 94
Operation Finished	5.9 (±5.8) 81	0.0 (±0.0) 85	6.0 (±5.7) 92	3.7 (5.7) 81	0 (0.0) 85	4.1 (5.8) 92

## Data Availability

The data presented in this study are available on request from the corresponding author. The data are not publicly available, as the open sourcing of all videos for general usage was not declared in the IRB approval.

## Appendix A

**Table A1:** RAPN phase list with the according visual cues.

	RAPN Phase	Starting Point	Visual Starting Cue	Phase Characteristics
1	Port Insertion and Surgical Access	Inside abdomen	When the video starts in the abdominal space or when the camera is inserted into the abdominal space and there is no visual of the trocar	Port placement; Instrument insertion; Adhesion removal
2	Colon (and Spleen) Mobilization	Identification of the mesenteric line A*: spleen mobilization	Right kidney: when the camera points between the liver and the right paracolic gutter Left kidney: when the camera points between the paracolic gutter and the kidney contour can be seen A* when instruments mobilize the spleen	Opening of peritoneum; Adhesion removal
3	Hilar Control General	Identification of gonadal vein A*: start dissection of renal vein	Identification of gonadal vein: when the gonadal vein can be partially seen A* when the renal vein can be partially seen	Dissection of main renal artery; Start isolation of main renal artery **; Vessel loop on main renal artery; Hemolock clip on vessel loop
4	Selective Hilar Control	Start dissection of other than main renal artery	When the artery can be partially seen	Dissection of selective renal artery; Start isolation of selective renal artery **; Vessel loop on selective renal artery; Hemolock clip on vessel loop
5	Kidney Mobilization	Start opening Gerota’s fascia	When a new incision in the perirenal fat, representing the surrounding renal fascia (Gerota’s fascia), is made	Dissection of kidney surface
6	Tumor Identification	Start demarcation of tumor A*: ultrasound start	When the monopolar curved scissors use coagulation to delineate tumor position whilst remaining on the kidney surface A* when the ultrasound probe is pressed against the parenchyma and the ultrasound image can be seen	Use of ultrasound probe; Tumor demarcation
7	Hilar Clamping	Bulldog clamp on main artery A*: bulldog clamp on selective artery	When the bulldog clamp is positioned on the renal artery When the bulldog clamp is positioned on a first- or higher-order renal artery	Use of bulldog clamp; Use of ICG
8	Tumor Excision	Start blunt dissection	When the monopolar curved scissors’ cut function is used to cleave open the renal parenchyma, or the tumor is being separated from the renal parenchyma whilst the scissor blades are closed	Resection of tumor
9	Specimen Retrieval	Free tumor base	When the tumor base is completely free from the tumor	Specimen retrieval
10	Inner Renorrhaphy	First inner renorrhaphy stitch	When the needle (not the needle wire) has fully exited the parenchyma after its first bite	Inner renorrhaphy
11	Hilar Unclamping	Bulldog clamp off main artery A*: bulldog clamp off selective artery	When the bulldog clamp is opened on the main renal artery A* when the bulldog clamp is opened on the selective artery	Hilar unclamping
12	Outer Renorrhaphy	First outer renorrhaphy stitch	When the needle (not the needle wire) has fully exited the parenchyma after its first bite	Outer renorrhaphy; Use of hemostatic agent
13	Specimen Removal and Closing	Tumor bagging	When the tumor falls halfway into the endobag	Tumor bagging; Use of hemostatic agent
14	Instrument Removal	Vessel loop removal A*: bulldog clamp removal	When the assistant fully grasps the already-cut vessel loop using a laparoscopic instrument When the assistant fully grasps the bulldog clamp using a laparoscopic instrument	Instrument removal
15	Retroperitonealization of the Kidney	Passing first stitch in parietal peritoneum	When the needle (not the needle wire) has fully exited the fatty tissue after its first bite	Suturing parietal peritoneum
16	End of Operation	Tension hem-o-lok retroperitonealization A*: secure suture retroperitonealization **	When the non-absorbable clip is pressed against the peritoneum while the needle wire is still under tension A* When the last clip is fully applied to the suture wire	Removal of robotic instruments **; Drain placement; Camera out of body; Camera stop

A*: Alternative starting point: when an alternative starting point occurs first, this will be the starting point of the phase. ** Alternative starting point only annotated when the typical starting point does not occur (also describes the beginning of the phase).
